# The changing role of Women in Food Microbiology: the case history of wine microbiologists in Italy

**DOI:** 10.3389/fmicb.2023.1217385

**Published:** 2023-06-22

**Authors:** Tiziana Nardi, Patrizia Romano

**Affiliations:** ^1^CREA - Council for Agricultural Research and Economics, Research Centre for Viticulture and Enology, Conegliano, Italy; ^2^Faculty of Economy, Universitas Mercatorum, Rome, Italy

**Keywords:** wine microbiology, gender equality, vineyard microbiology, viticulture and oenology research, food microbiology

## Introduction

Wine production has a long history, dating back well over 7,000 years. Scientific knowledge has grown at an exponential rate, and historical milestones in chemistry and biology have shaped our understanding of the biology of microorganisms that drive fermentation.

Chemists, not biologists, made the first scientific studies of alcoholic fermentation and the first evidence of understanding this phenomenon dates back to 1789 in “Elementary chemistry treatise,” where the famous French chemist Antoine-Laurent de Lavoiser described wine fermentation as a chemical reaction of grape must in carbonic acid and alcohol. He was the first to write a chemical reaction as an equation. Subsequently in 1815, another great French chemist, Joseph Gay-Lussac, revised the chemical stoichiometry of alcoholic fermentation, formulating the mathematical relationship that governed the transformation of sugars into alcohol and carbon dioxide.

In the fascinating journey from the vineyard to the cellar, the production of wine is a complex process where microorganisms play a fundamental and decisive role. In fact, the first microbiologists, such as Pasteur and Müller-Thurgau, observed the presence of microorganisms in wine, and the importance of microorganisms in winemaking was beginning to be understood.

In 1857 Luis Pasteur published “Mémoire sur la fermentation alcoolique,” which marked the beginning of a growing fascination for the biology of wine microorganisms among researchers all over the world, representing a milestone in the history of science and its applications. It's merit of Pasteur, unanimously considered the founder of wine microbiology, to have demonstrated experimentally and irrefutably the microbiological nature of both fermentation and wine diseases. In particular, Pasteur (1858) postulated that fermentation is a process producing energy for microorganisms, such as bacteria and yeasts under anaerobic conditions and associated the breakdown of sugar to alcohol and carbonic acid with the living processes. His studies demonstrated the essential role played in this process by yeasts, i.e. that the production of alcohol was due to the development of yeasts (Pasteur, 1860).

In 1890 Hermann Müller-Thurgau introduced the concept of inoculation of wine, fermented with selected pure ferments and in 1891 he demonstrated that bacteria are responsible for malolactic fermentation.

Other scientists in the late 1800s and early 1900s demonstrated the importance of inoculating selected cultures, such as Robert Koch (1900), who demonstrated that inoculation with malolactic bacteria (*Oenococcus oeni*) can reduce the acidity of wine (malolactic fermentation) and Emil Hansen (1888), who perfected Pasteur's method for isolating pure yeast cultures (for a review, see Chambers and Pretorius, 2010). In the 1960s, the Italian microbiologist Tommaso Castelli demonstrated the existence of a link between yeast and the viticultural environment and isolated the different yeast species from spontaneous fermentations, already underlining the important role of non-*Saccharomyces* yeasts, especially apiculate yeasts.

The progress of wine microbiology up to the mid-1900s was developed by famous scientists of chemical and biological sciences, who laid the foundations for the knowledge and understanding of the phenomenon of the transformation of grape must into wine. These eminent scientists were all men and before finding a woman cited for topics concerning the microbiology of wine one must go to the end of the 1970s with Linda Bisson's studies on metabolism of the yeast *Saccharomyces cerevisiae* and Aline Lonvaud-Funel's research on the malolactic fermentation.

Later on, the participation of women in the scientific field of wine microbiology can be told by several evidence. This testifies their contribution to the development, diffusion and transfer of knowledge in this fascinating discipline worldwide, even though proofs are discontinuously reported up to the end of the last century. As a case study for analyzing gender balance in this subject, therefore, we decided to track back the involvement of female researchers in wine microbiology in Italy in the last 20 years, as attested both by their presence within the most eminent groups of experts in the field, and by their essays within the reference texts and handbooks published over time on the subject.

The aim of this short essay, in the context of the Frontiers Women in Food Microbiology collection (a dedicated Research Topic aimed at reveling the achievements of women in celebration of International Women's Day) is to unveil and promote female contribution in this field, looking toward gender equality, a significant matter since according to UNESCO Institute for Statistics just 30% of the world's researchers in STEM are women.

## Membership organizations and scientific societies dealing with Food and Wine Microbiology in Italy—A look on gender balance over time

**Figure 1:**
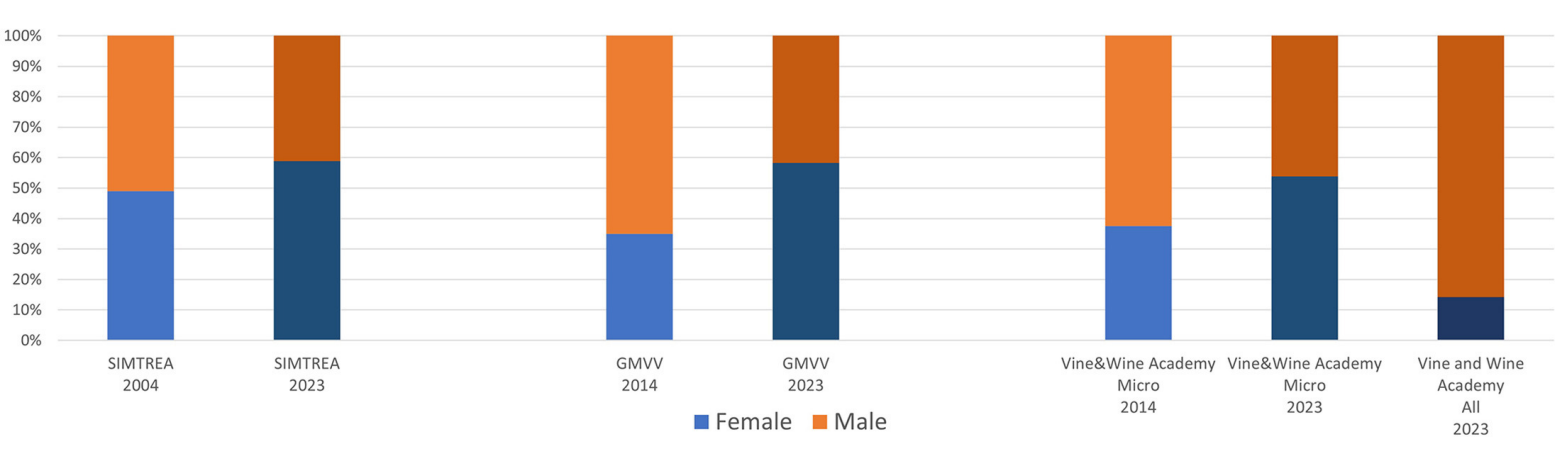
Gender balance over time in different scientific groups.

The Italian Society of Food, Agricultural and Environmental Microbiology (SIMTREA) is a nonprofit membership organization for scientists who work in the fields of agricultural, environmental and food microbiology, established in 1994 in Milan (Italy). It has members coming from universities, industry and research institutions [Italian Society of Agro-Food and Environmental Microbiology (SIMTREA), 2023]. The Society promotes the understanding of microbiology to a diverse range of stakeholders, including policy makers, students, and teachers. SIMTREA is a member Society of FEMS, The Federation of European Microbiological Societies. Looking at its members at a glance, we can recognize that almost 20 years ago (2004), SIMTREA counted 102 associate members, of which 52 males and 50 females (49%). Over time, not only the number of associated scientists in agri-food microbiology has strongly increased (to date this society counts 296 members), but also the gender balance has changed, getting up to 59% of females (174, vs 122 males), as reported in Figure 1.

For examining more thoroughly the gender balance within researchers actively involved in wine microbiology, we can get some interesting information from a subsection of SIMTREA: the Microbiology of Vine and Wine Group (GMVV). The GMVV was established in 2014, under the coordination of a woman, to bring together the expertise of the national research groups (Universities/Institutions) operating in the wine microbiology sector (GMVV, 2014). The GMVV addresses many issues professors hold this apical concerning microorganisms starting from the vineyard to the wine product and throughout the fermentation process, promotes collaborations in the research sector and the transfer of knowledge, organizes scientific and informative meetings, organizes joint experiments aimed at validating microbiological protocols. When established, in 2014, the Group counted 20 members from 20 research groups, out of which 7 (35%) were women. Today, in 2023, this group has also grown and changed its gender balance, as it counts 55 members from 28 research groups, out of which 32 (58%) are women (Figure 1).

Another interesting cross-cutting aspect among disciplines, is that vine and wine microbiology is strictly connected with all the other research sectors involved in the study of vine and wine, spanning from viticulture and grapevine genetics to oenology and wine chemistry, or to sensory analysis. In this field, a very ancient and well renowned institution in Italy is the Italian Academy of Vine and Wine (“Accademia Italiana della Vite e del Vino”). Established in Siena in 1949, with the intention of creating a group of experts capable of promoting the progress of Italian vitiviniculture, it rapidly got the applause and encouragement of eminent government officials and distinguished Italian and foreign scholars. Nowadays, the Academy counts overall 218 distinguished members (Accademia Italiana Della Vite e Del Vino, 2023). Among them, 13 are microbiologists, representing the vine and wine microbiology expertise within the Academia. At present, women are well represented among this expert sub-group, being 54% of the microbiology members (seven out of 13), with participation also increasing over time (as reported in Figure 1). It is worth to note, though, that this is not at all the case when considering the whole Academy, where gender balance is far to be reached, since only 14% (31) of the 218 full members are women. Therefore, if we consider all the different skills linked to vitiviniculture and represented within this eminent association, the road to achieving a balanced female presence is still long: at the same time, nonetheless, the example of the wine-microbiologists subgroup can be taken as example of a success story.

## Reference books on Wine and Vine Microbiology—The contribution of female experts over time

Up to the end of the 20th century, reference books for studying wine microbiology in Italy were written by a single or few eminent authors, which were all men. This is the case, for instance, of old (and presently out of stock) books as “Treaty of wine microbiology” (Verona and Florenzano, 1956), or “Techniques of wine Microbiology” (Delfini, 1982), or again of “Wine Microbiology and Biotechnology” (Zambonelli, 1988). These milestone-books were a reference both for students (at agricultural schools and universities) and for winemakers and technicians involved in wine production. All the authoring mentor figures, therefore, at that time were men.

It is only since 2005 that the first contribution of female-experts did appear, both as editors and authors in newly concepted textbooks, written thanks to the contribution of several authors, under the coordination of first-rate curators. The last 20 years, therefore, have marked an important change for the contribution of women in wine microbiology, also from the important point of view of knowledge transfer. The book “Wine Microbiology” (Vincenzini et al., 2005), firstly edited in 2005 by Vincenzini, Romano and Farris, hosts a woman within the three curators, and 21 women (51%) among the 41 authors. Some years later, in 2014, a book entirely curated by women was published [“Oenological Microbiology” (Suzzi and Tofalo, 2014), edited by Suzzi and Tofalo], which hosted the contribution of 16 female authors (62%) out of 26 total ones. Last but not least, the recent publication “Vine and Wine Microbiology” (Romano et al., 2022), edited by Romano, Ciani and Cocolin in 2022, hosts 25 women (51%) within its 49 authors and still one female curator out of 3. These data show the stable and important presence of renowned and experienced women among the experts giving their contribution to Wine Microbiology books over the last 20 years, testifying again how this discipline shows nowadays a well-defined gender balance.

## Discussion: a success story but with room for improvement

However, there is still some way to go for achieving full gender equality, even in a discipline that can be considered as a “success story” for the presence of women such as, in our opinion, wine microbiology (as far as can be extrapolated from the Italian case study). With Horizon Europe, indeed, the European Commission affirms its commitment to gender equality in research and innovation [Directorate-General for Research Innovation (European Commission), 2021], and includes pay equality and removing pay gaps for Women in STEM within its goals. Indeed, the legal base sets gender equality as a crosscutting priority and establishes strengthened provisions, but the gender pay gap in the EU stands at 12.7% in 2021 and has only changed minimally over the last decade (this means that women earn 13.0% on average less per hour than men). From this point of view, if we analyze the positioning data of academic staff (including researchers, assistants/associate professors and full professors in universities) in the field of Agri-food Microbiology in Italy, we can observe that only 18 of the 103 enrolled women (17%) are full professors, while 35% (27 out of 77) of male professors hold this apical career role (CINECA, 2023).

It has therefore to be mentioned that, although women are satisfactorily represented in Food and Wine Microbiology in Italy, thus representing a success story compared to other disciplines (in particular, those broadly associated to Viti-viniculture), female scientists still have difficult times to see their adequate representation in the highest-level roles, due to some gender disparities. Although these disparities were much heavier for the former generation of female wine microbiologists (as told by numbers about the situation 20 years ago, Figure 1) and have been successfully overcome in a major part by the effort and competences of women microbiologists over time, they still somehow exist. Since science and gender equality are essential to ensure sustainable development, as highlighted by UNESCO, we therefore believe that initiatives to promote the work of female researchers and highlight the diversity of research performed across the entire breadth of Food and Wine Microbiology have still room for being effective, in order to complement local and global governmental policies for improving gender equality.

## Author contributions

TN and PR contributed to conception and writing of the Opinion. All authors contributed to manuscript revision, read, and approved the submitted version.
